# 2257: Vaccine efficacy and immunogenicity of recombinant WAP and CAP-1 proteins in AKR mice

**DOI:** 10.1017/cts.2017.215

**Published:** 2018-05-10

**Authors:** Neima Briggs, Leroy Versteeg, Bin Zhan, Rojelio Mejia, Brian Keegan, Coreen Beaumier, Jagannadha Sastry, Maria Elena Bottazzi, Peter Hotez

**Affiliations:** Baylor College of Medicine, Houston, TX, USA

## Abstract

OBJECTIVES/SPECIFIC AIMS: *Trichuris trichiura*, is a leading cause of chronic colitis worldwide, resulting in growth stunting, anemia, and cognitive deficits, predominately in children. Our long-term goal is to develop a vaccine against *T. trichiura*. Both *T. trichiura* and the closely related *Trichuris muris* release excretory/secretory (ES) macromolecules from the stichosome organ, which facilitates intracellular invasion into the cecum. We exploited the high degree of genetic sequence homology between *T. trichiura* and *T. muris* to evaluate *T. muris* stichosome proteins as vaccine candidates. METHODS/STUDY POPULATION: Through immunological screening of the *T. muris* stichosome cDNA library and *T. muris* ES macromolecules generated in culture, we identified, cloned, and expressed several vaccine candidates. In ranking these antigens, we selected the most promising recombinant proteins, WAP and CAP-1, and vaccinated AKR mice to evaluate the immunogenicity and efficacy of our candidate. In addition, we collected 240 serum samples in the *T. trichiura* endemic region of Honduras to measure the recognition of WAP and CAP-1 in naturally infected human. RESULTS/ANTICIPATED RESULTS: We measured a statistically significant reduction in larval worm burden in WAP and ES vaccinated mice, but not CAP-1, by microscopy and real-time PCR of *T. muris* DNA. We found a significant relationship between antigen-specific IgG1:IgG2a ratio in the mouse serum and degree of protection. Mouse splenocytes, vaccine-sensitized draining lymph nodes, and mesenteric lymph nodes were antigen-stimulated and their secreted Th1, Th2, and Th17 cytokines were measured by Luminex^®^. Stimulated mouse lymphoid cells were further analysed for T helper, T cytotoxic, and central/effector memory T cells by Flow Cytometry. Human IgG1, IgG2, and IgE antibody titers against WAP and CAP-1 were measured by ELISA. DISCUSSION/SIGNIFICANCE OF IMPACT: In our study, we describe the T cell dependent mechanism of humoral immunity of 2 promising ES-derived vaccines recombinant proteins, WAP and CAP-1. We evaluated the immune response, indicating a driving a Th2-induced humoral response necessary for protection. We further predict protection and allergenicity of WAP in humans using serum from a cohort in an *T. trichiura* endemic region.

